# Immunological characterization of IgG subclass deficiency reveals decreased Tregs and increased circulating costimulatory and regulatory immune checkpoints

**DOI:** 10.3389/fimmu.2024.1442749

**Published:** 2024-08-14

**Authors:** Per Wågström, Maria Hjorth, Daniel Appelgren, Janne Björkander, Charlotte Dahle, Mats Nilsson, Åsa Nilsdotter-Augustinsson, Jan Ernerudh, Sofia Nyström

**Affiliations:** ^1^ Department of Infectious Diseases, Ryhov County Hospital, Jönköping, Sweden; ^2^ Division of Inflammation and Infection, Department of Biomedical and Clinical Sciences, Linköping University, Linköping, Sweden; ^3^ Division of Diagnostics and Specialist Medicine, Department of Health, Medicine and Caring Sciences, Linköping University, Linköping, Sweden; ^4^ Wetterhälsan Primary Health Care Centre, Region Jönköping County Jönköping, Sweden; ^5^ Futurum – the Academy for Health and Care, Region Jönköping County, Sweden; ^6^ Department of Health, Medicine and Caring Sciences, Linköping University, Linköping, Sweden

**Keywords:** predominantly antibody deficiency, IgG subclass deficiency, Ig replacement therapy, T cells, B cells, Tregs, immune checkpoints

## Abstract

**Background:**

Immunoglobulin G subclass deficiencies (IgGsd) comprise a wide clinical spectrum from no symptoms to repeated respiratory infections and risk for the development of lung damage. Our aims were to investigate whether the immunological phenotype of IgGsd patients on and off immunoglobulin replacement therapy (IgRT) was reflected in the clinical features of IgGsd.

**Method:**

Thirty patients with IgGsd were included in this prospective study of 18 months of IgRT, followed by 7-18 months of IgRT discontinuation. Blood samples were collected when patients were on and off IgRT and compared with samples from 34 cross-sectional healthy controls. An in-depth lymphocyte phenotyping was performed by flow cytometry and plasma levels of immune checkpoints were assessed.

**Results:**

IgG3 subclass deficiency was most common. Patients with IgGsd had decreased levels of activated T cells and B cells and plasma levels of negative immune checkpoint molecules correlated negatively with T cell and B cell activation. The decreased T cell activation level was unaffected by IgRT, while the B cell activation was partly restored. Of note, decreased levels of activated regulatory T cells (Tregs) were found in IgGsd patients and was partly restored during IgRT. The profile of comorbidities did not associate with Treg levels.

**Discussion:**

IgGsd is associated with decreased B cell and T cell activation including Tregs, and increased plasma levels of negative immune checkpoint molecules. The consequence of reduced activated Tregs in IgGsd remains unclear. Decreased immune cell activation was partly restored during IgRT, demonstrating that IgRT may contribute to improved immune function in patients with IgGsd.

## Introduction

Predominantly antibody deficiencies (PAD) comprise a group of inborn errors of immunity with poor antibody responses (1). PAD is associated with increased susceptibility to infections and with chronic inflammatory disorders (2). Immunoglobulin G subclass deficiency (IgGsd) is a mild form of PAD characterized by increased frequencies of infections, and reduced levels of at least one IgG subclass. According to the international classification, which aligns with Swedish classification, IgGsd characterized by reduced levels of least one of IgG subclass and is mostly an asymptomatic PAD, but IgGsd can also be associated with increased frequencies of infections. IgGsd can be associated with subnormal levels of IgA or moderately reduced IgG (1). According to the European Society of immunodeficiency, IgGsd should be accompanied by normal levels of IgG, IgM and IgA otherwise it is considered an unclassified antibody deficiency (3). The clinical presentation of IgGsd varies from asymptomatic to recurrent infections and symptomatic IgGsd is often associated with atopic disease and chronic lung disease (4–6). Bronchiectasis, affect 38%-48% of patients with IgGsd. Recurrent airway infections may contribute to airway remodeling and deteriorated lung function in patients with IgGsd (7–9). Autoimmune conditions are also frequent among patients with IgGsd. The prevalence of autoimmune conditions, such as rheumatoid arthritis, thyroiditis and Sjögren syndrome, was over 40% in a large cohort of patients with IgGsd (10). Furthermore, in a study on inborn errors of immunity, rheumatologic complications were most common in patients with IgGsd (11%) when compared to other groups of patients with predominantly antibody deficiencies (11). Hence, low IgG subclass levels may reflect an underlying immune dysregulation, but the understanding of factors contributing to autoimmune complications in IgGsd is scarce.

To date, there is no pathogenic gene variant associated with IgGsd, or with any other PAD characterized by reduced IgG levels not fulfilling the criteria of common variable immunodeficiency (CVID). Common variable immunodeficiency (CVID) refers to a group of heterogenic severe PAD characterized by significant hypogammaglobulinemia, in combination with perturbations of circulating B cell and T cell subsets (12). In CVID an increasing number of pathogenic gene variants have been reported (1). As in unspecified PAD and IgGsd, autoimmunity and lung disease affect many patients with CVID (13). In contrast to IgGsd, lymphoproliferation (i. e polyclonal lymphocytic infiltration of non-lymphoid tissues) may complicate CVID, and CVID is associated with markers of more severe immune defects than IgGsd (14).

Patients with CVID are characterized by decreased class-switched memory B cells (15) and low regulatory T cells (Tregs) associates with autoimmune manifestations in CVID (16). There is, to our knowledge, there is only one previous report on circulating lymphocyte subsets, e.g., B cells, CD4^+^ helper (h) T cells, CD8^+^ cytotoxic T cells and natural killer (NK) cells, in patients with IgGsd. In a study of 16 patients with IgG2 deficiency, the only anomaly found among lymphocytes, was subnormal levels of Th cells in one patient (10). In the vast majority of the patients in a cohort of 17 patients with IgG3 deficiency, there were normal numbers of, or close to normal numbers, of B cells, Th cells, cytotoxic T cells and NK cells (17). Notably, altered functional T cell responses were reported in occasional patients in this restricted cohort, which may reflect T cell subpopulation perturbations. Decreased switched memory B cells have been reported in unspecified IgG deficiency (14) and it can be hypothesized that the numbers of class-switched memory cells are informative about the underlying immune response also in IgGsd. Impaired infection control may also result in perturbation of the T cell compartment. Accumulating data support a role for Tregs in various autoimmune diseases (18). Given the high prevalence of autoimmunity in IgGsd, data on the frequencies of Tregs could increase the understanding of factors contributing to autoimmunity in patients with IgGsd.

Immunoglobulin replacement therapy (IgRT) consists of monomeric IgG pooled from healthy donors which is administrated subcutaneously or intravenously and aims to improve the immune response to pathogens in patients with PAD. High dose intravenous immunoglobulin therapy, on the other hand, is used to alleviate inflammatory conditions by immune modulatory mechanisms. In patients with IgGsd, IgRT reduces bacterial infections and improve the quality of life (6, 17, 19) and IgRT should be considered in patients with clinically significant infections (20). Consensus on the use of IgRT in IgGsd and other minor PAD is lacking, and it is essential to define reliable predictors identifying the subgroup of patients with IgGsd that benefit from IgRT. In Sweden, the decision to introduce IgRT is based on the frequency and severity of bacterial infections and the presence of any lung damage. According to present Swedish national guidelines (21), the effect of IgRT should be evaluated after 12-18 months and followed by a discontinuation trial, during which IgRT is reintroduced if clinically significant infections reoccur or if there are signs of lung function deterioration when IgRT is discontinued. It has been suggested that immunoglobulins used in replacement therapies may modulate the immune response (22, 23), however to our knowledge there is only one *in vivo* study on the long term immune-modulatory effects of IgRT in CVID (24). Longitudinal prospective studies of the immune modulatory effects of IgRT in hypogammaglobulinemia are important for the understanding of the pathological processes.

In this study, we performed in-depth characterization of the B cell and T cell compartments of a clinically well-characterized cohort of patients with IgGsd. Plasma levels of costimulatory and regulatory immune checkpoint molecules were also evaluated. In addition, any changes in subsets of B cells, T cells and the levels of costimulatory and regulatory immune checkpoints in patients with IgGsd during IgRT were investigated. We found that IgGsd was associated with decreased levels of activated T cells and B cells and that plasma levels of negative immune checkpoint molecules correlated negatively with T cell and B cell activation. IgGsd was also characterized by decreased levels of activated regulatory T cells (Tregs), which were partly restored during IgRT.

## Material and methods

### Ethics

The study protocol was approved by Regional Ethical Review Board in Linköping, Sweden (Dnr 2011/506-31). Written informed consent was obtained from all study participants.

### Study population and design of the study

This prospective study was conducted at two Swedish regional centers of infectious disease during 2012-2015. Of 85 adult patients diagnosed with IgGsd, 35 (22 women and 13 men) fulfilled the inclusion criteria (no severe lung disease and no previous IgRT discontinuation trial). The cohort has previously been described (25). Patients kept track of their infections during the study period and blood samples were collected after 18 months on IgRT, and at least seven months after discontinuation of IgRT. Samples both when on and off IgRT were available for thirty patients (19 women and 11 men). Thirty-four healthy blood donors (15 men and 19 women, median aged 54, range 28-68 years), served as cross-sectional controls. The control population was in good physical health. Three controls (all 62 years or older) reported regular use of antihypertensive drugs and another control was on amitriptyline for neuropathic pain.

### Handling of blood samples

Blood samples were drawn in vacutainers, and immune phenotyping were performed on whole blood. Levels of IgG, IgA, IgM, and IgG subclasses IgG1, IgG2 and IgG3 were measured according to standard operating procedures at the Laboratories of Clinical Chemistry in the Counties of Jönköping and Östergötland. Plasma samples were stored at -80°C until plasma protein profiling.

### Lymphocyte phenotyping with flow cytometric analysis

Absolute T-, B- and NK-cell numbers were determined by flow cytometry with the use of BD Multitest™ reagent (BD Biosciences; 340499 and 340500). Briefly, antibodies were added to fresh EDTA whole blood and erythrocytes were lysed with BD FACS™ lysing solution (BD Biosciences). Data were acquired with BD FACSCanto™ II (BD Biosciences) flow cytometer. Phenotyping of T- and B-cell subsets was performed in 7- or 8-color combinations. The mAbs used are listed in (**Supplementary Table S1**). Kaluza flow cytometry software version 1.5 (Beckman Coulter, Miami, USA) was used for data analysis. Identification of lymphocytes was performed by CD45 and side scatter (SSC) or forward scatter (FSC) and SSC. HLA-DR was used as a marker for activation of CD4^+^ helper and CD8^+^ cytotoxic T cells. Differentiation status of CD4^+^ T helper and CD8^+^ cytotoxic T cells was determined with CD45RA and CCR7. Regulatory T cells (Tregs) were defined as resting (CD45RA+CD25dim) or activated (CD45RA-CD25bright) T helper cells. Subtyping of naïve and memory B cells was determined in CD19^+^ cells using CD27 and IgD. Further subtyping of B cells was performed using CD21, CD24, CD25, CD38 and IgM.

### Plasma protein profiling

Frozen plasma samples were sent to the Clinical Biomarkers facility, Science for Life Laboratory, Uppsala University (Uppsala, Sweden) and analyzed there according to their standard operating procedures. Proseek multiplex assay inflammation panel (95302) was used for detection of 92 different inflammatory associated proteins (Olink Proteomics, Uppsala, Sweden). Of 81 proteins detected in at least 40% of samples, 19 had a predominantly plasma membrane location and were selected for further investigation (**Supplementary Table S2**).

### Statistical analyses

For comparisons between two groups, Mann-Whitney U-test was used, or the Wilcoxon test for paired samples, and ANOVA with Tukey post-test if parametric data. Statistical significance was defined as p<0.05. SAS/STAT^®^ ver. 13.1 software (Copyright ^©^ 2002-2012 by SAS Institute Inc., Cary, NC, USA), Statistica Ver.13, (Copyright ^©^ 1984-2017, TIBCO Software Inc., CA, USA), IBM SPSS Statistics 28 (SPSS inc., IL, USA) and GraphPad Prism 9.3.1 (GraphPad software, CA, USA) were used for the calculations. GraphPad Prism was used for graphics. Bioinformatics & Evolutionary Genomics was used for the construction of Venn-diagrams (26).

## Results

### Characteristics of the study cohort

**Figure 1:**
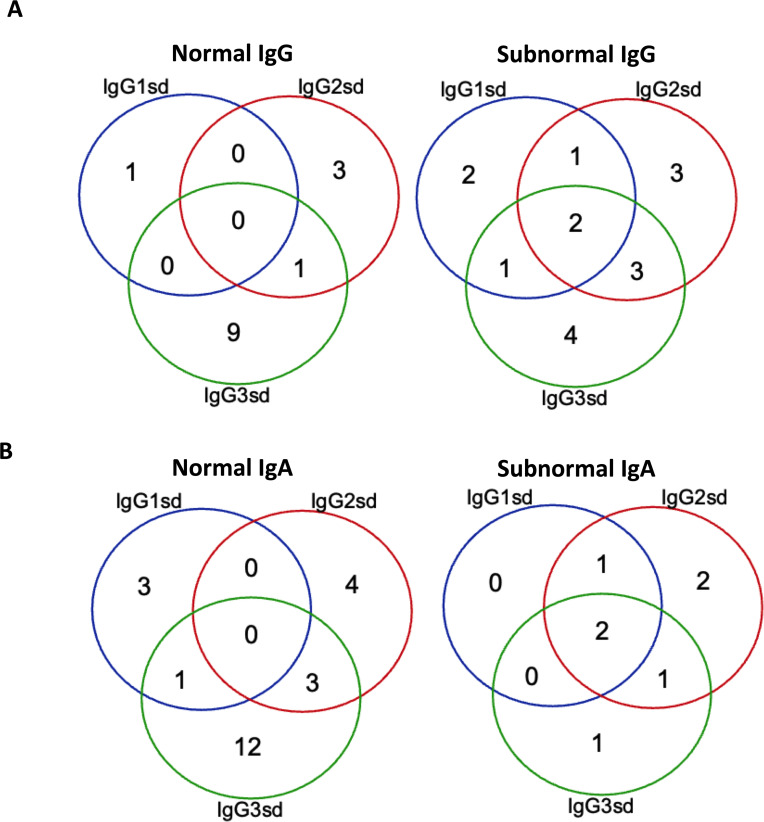
Isolated IgG3 subclass deficiency most common among patients with normal IgG. Venn-diagram showing the distribution of single and combined IgG-subclass deficiencies related to plasma levels of IgG **(A)** and IgA **(B)** in patients with IgGsd (n=30). Quantitative detection of serum immunoglobulins was performed by routine analyses using validated techniques.

The group of IgGsd patients was heterogeneous, with diverse types of comorbidities (**Table 1**), and has previously been described in detail (25). The most common associated comorbidities were autoimmunity and lung disease. Seven patients were affected by both autoimmunity and lung disease. Ten patients were considered having a profession associated with an increased risk of exposure to airway pathogens, e. g preschool teacher and healthcare worker. Sixteen patients had subnormal IgG levels and seven patient subnormal IgA levels (**Figures 1A, B**). Subnormal IgA or IgG was any concentration below the lower reference limits referring to the 2.5 percentile of a healthy Swedish population (27). IgG3 deficiency was present in ten of the 14 patients with normal levels of total IgG and in 16 of 23 patients with normal levels of IgA. IgG1 deficiency and mixed IgGsd were most common in the group of patients with subnormal IgG levels (**Figure 1A**). Six patients of the seven patients with subnormal IgA had IgG2 deficiency (**Figure 1B**). Subnormal IgM levels were found in five patients (results not shown).

**Table 1:** Demographics, comorbidities, and Ig levels in patients with IgG subclass deficiency.

	Controls (n=34)	Patients with IgGsd (n=30)
Women, *n* (%)	19 (56)	19 (63)
Age, median years (range)	54 (28-68)	58 (20-72)
Risk profession, *n* (%)	–	9 (30)
Subnormal lung function test, *n* (%)^a^	n/a	6 (20)
Lung disease, *n* (%)	n/a	13 (43)
Bronchiectasis, *n* (%)	n/a	9 (26)
Ever smoke, *n* (%)	–	8 (23)
Atopy, *n* (%)	–	9 (30)
Autoimmunity, *n* (%)	n/a	16 (53)
Heredity for autoimmunity, *n (%)*	–	6 (20)
Ig-levels, g/L, *median (IQR)* ^b^		n=30
IgM	–	0.79 (0.30-0.93)
IgA	–	1.5 (0.84-2.1)
IgG	–	6.2 (4.9-8.6)
IgG1	–	3.6 (2.7-5.3)
IgG2	–	1.8 (0.61-2.6)
IgG3	–	0.15 (0.10-0.4)

Reference values (g/L); IgM 0.27-2.1; IgA 0.88-4.5; IgG 6.7-15; IgG1 2.80-8.0; IgG2 1.15-5.7; IgG3 0.24-1.30.

a FEV1<80%; ^b^Without Ig-replacement therapy; -, information not available; IQR, inter-quartile range.

### Circulating lymphocyte populations are unaffected by IGRT in IgGsd

To evaluate if patients with IgGsd had phenotypic abnormalities in their lymphocyte populations, a broad flow cytometry assessment was performed with paired samples collected when on and when off IgRT. Samples collected from patients were compared to 34 cross-sectional healthy controls. The absolute numbers and percentages of major lymphocyte populations (CD4^+^ and CD8^+^ T cells, B cells and NK cells) were similar in patients with IgGsd and in the controls (**Table 2**). The major lymphocytes populations, e.g., Th cells, cytotoxic T cells, B cells and NK cells, were not affected by IgRT since levels were similar when on and off treatment.

**Table 2:** Lymphocyte populations in patients with IgG subclass deficiency and controls.

	Controls		IgG subclass deficiency			
			All off IgRT		All on IgRT	
	(n=34)		(n=30)		(n=30)	
**Subset**	**Median**	**IQR**	**Median**	**IQR**	**Median**	**IQR**
Lymphocytes x10^9^/L	1.7	1.40-2.2	2.0	1.4–2.5	1.8	1.4-2.5
% of leukocytes	31	26–35	28	23–35	30	27–36
T-cells CD3^+^/µL	1268	1019-1733	1405	1041–1968	1364	968-2055
% of lymphocytes	76	71–80	73	66–80	73	66–79
T-helper CD3^+^4^+^/µL	800	702-929	927	615–1089	858	539–1404
% of lymphocytes	46	39-53	43	37–53	44	36-49
T-cytotoxic CD3^+^8^+^/µL	383	310-645	452	355–663	513	329–620
% of lymphocytes	24	20-34	26	18–32	23	20-30
B-cells CD19^+^/µL	182	130-221	221	124–355	184	118–350
% of lymphocytes	10	7.1-14	12	7.7–14	11	7.4-14
NK-cells CD16^+^56^+^/µL	217	151-345	226	149–403	259	172-391
% of lymphocytes	13	11-17	15	8.5–20	14	9.6-14

IgRT, Ig replacement treatment; IQR, inter-quartile-range.

### Alterations in IgGsd patients’ B cell subsets normalized during IgRT

**Figure 2:**
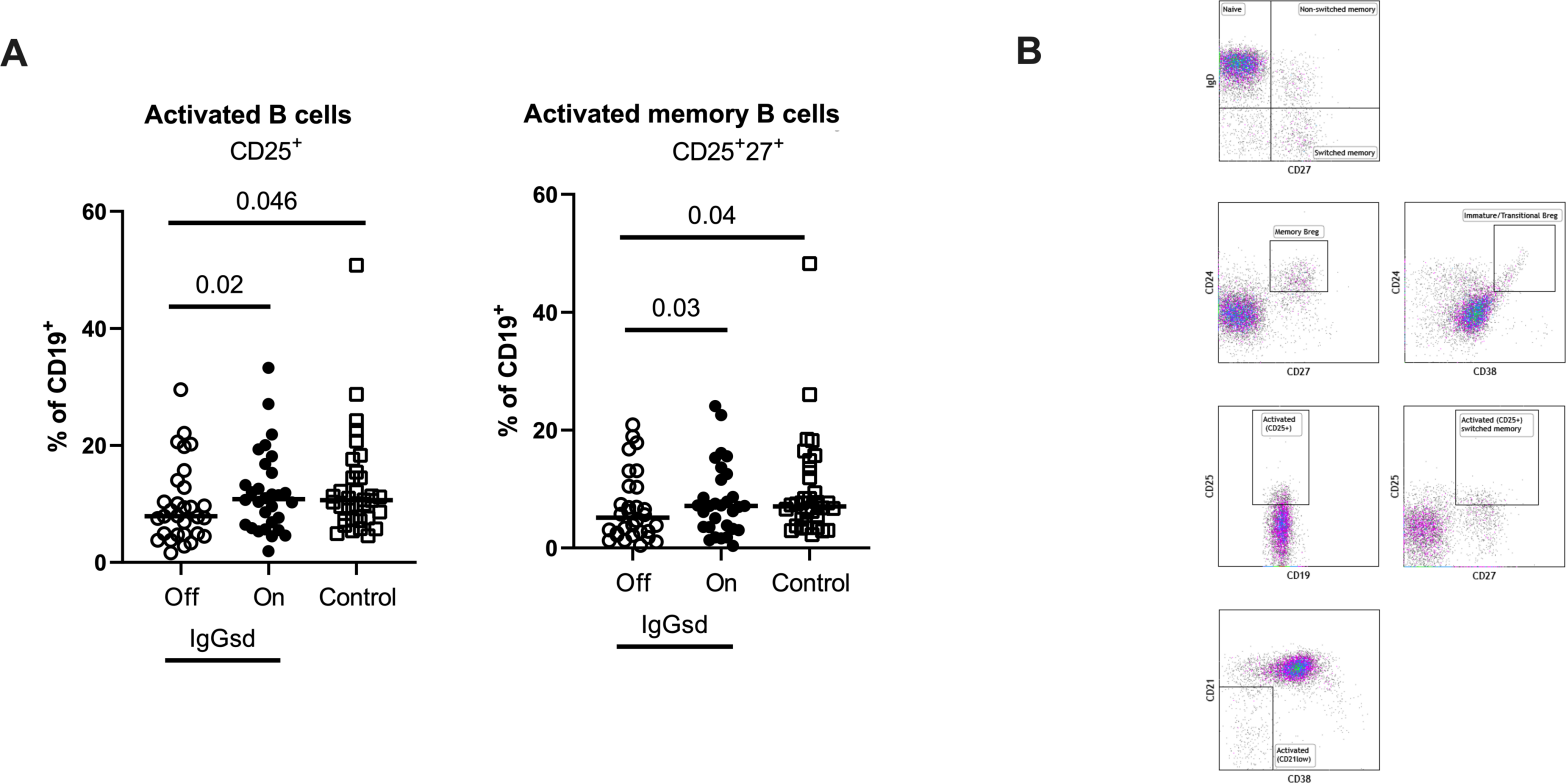
Ig-subclass deficiency was associated with decreased activation of B cells. Frequencies of activated and activated memory B-cells in Ig-subclass deficiency (IgGsd) patients when off (open circle) and on (filled circle) Ig-replacement therapy (IgRT) in relation to healthy controls (open squares) were determined by flow cytometry **(A)**. Definition and gating strategies of CD19^+^ B cell subsets **(B)**. Wilcoxon paired test was used for comparisons between IgGsd patients off and on IgRT, while Mann-Whitney U-test was used for non-paired analysis. Breg, regulatory B cells.

A deeper characterization of the B cells revealed aberrations restricted to the CD25^+^ B cell subsets in patients off IgRT compared to controls (**Table 3**). The proportions of activated CD25^+^ B cells and activated memory CD25^+^ CD27^+^ B cells were lower in IgGsd when off IgRT, while levels on treatment were normalized and did not differ from controls (**Figure 2**). Activated CD21^Low^ and switched memory IgD^-^ CD27^+^ B cells were similar across groups. In summary, patients with IgGsd had aberrant activation of B cell subsets that were normalized during IgRT.

**Table 3:** B cell subpopulations in patients with IgG subclass deficiency.

	Controls		IgG subclass deficiency			
			All off IgRT		All on IgRT	
	(n=34)		(n=30)		(n=30)	
% of B cells	Median	IQR	Median	IQR	Median	IQR
Naïve IgD^+^CD27^-^	60	47-71	59	48-79	60	47-81
Non-switched memory IgD^+^CD27^+^	6.3	3.2-9.3	6.8	3.2-12	6.5	3.2-14
Switched Memory IgD^-^CD27^+^	17	13-28	18	6.8-24	17	6.9-25
Immature/Transitional CD24^high^CD38^high^	3.9	2.5-4.9	3.5	2.8-5.6	3.9	2.8-4.9
Memory B reg CD24^high^CD27^+^	15	7.6-19	11.8	6.6-21	14	8.2-22
Activated CD21^low^	4.4	3.4-8.2	5.6	4.1-8.0	5.6	3.1-7.8
Activated CD25^+^	11	7.3-14	7.9*	4.8-13	11	6.5-15
Activated memory CD25^+^CD27^+^	7.1	4.9-12	5.2*	2.5-7.4	7.1	3.6-12

IQR, inter-quartile range; ^*^p<0.05, compared to controls. Wilcoxon paired test was used for comparisons between IgGsd patients off and on therapy, while Mann-Whitney U-test was used for non-paired analysis.

### Decreased T cell activation in IgGsd and increased CD8^+^ T cell differentiation during IgRT

**Figure 3:**
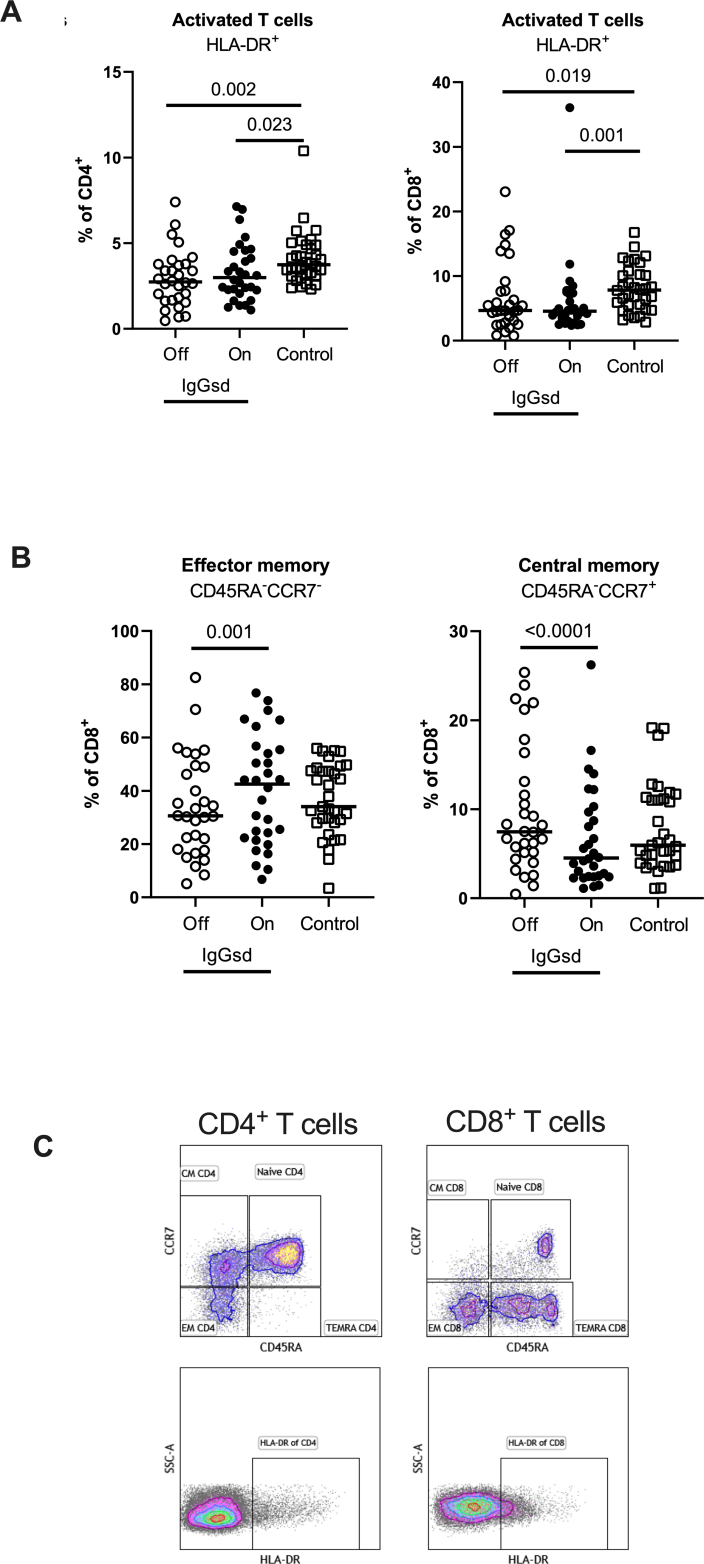
IgG replacement therapy is associated with increased CD8^+^ T cell differentiation in patients with IgG-subclass deficiency. Frequencies of activated CD4^+^ T-helper and CD8^+^ cytotoxic T cells **(A)**, effector memory and central memory CD8^+^ cytotoxic T cells **(B)** were assessed by flow cytometry in patients with Ig-subclass deficiency (IgGsd) when off (open circle) and on (filled circle) immunoglobulin replacement therapy (IgRT) and compared to healthy controls (open squares). Definition and gating strategies of naïve- memory- and activated subsets of CD4^+^ and CD8^+^ T cells **(C)**. Wilcoxon paired test was used for comparisons between IgGsd patients off and on immunoglobulin replacement therapy, while Mann-Whitney U-test was used for non-paired analysis. CM, central memory; EM, effector memory; TEMRA, terminally differentiated effector memory RA^+^.

Within the T cell compartment, we found decreased proportions of activated (HLA-DR^+^) CD4^+^ and CD8^+^ T cells in IgGsd patients off IgRT compared to controls (**Figure 3A**). The differences in T cell activation remained unaffected by IgRT. The proportions of terminally differentiated memory (TEMRA) CD4^+^ and CD8^+^ T cells were similar across groups, as well as the proportions of senescent (CD28^-^) CD4^+^ and CD8^+^ T cells, both when on and off IgRT (**Table 4**). On the other hand, IgRT induced a redistribution of memory cells within the CD8^+^ T cell population. When on IgRT, the proportions of CD45RA^-^CCR7^+^ central memory CD8^+^ cells were lower, while the proportions of CD45RA^-^ CCR7^-^ effector memory CD8^+^ T cells were higher compared with off-treatment samples (**Figure 3B**). Gating strategies for T cell subsets are shown in **Figure 3C**. Taken together, IgGsd was characterized by a decrease in activated T cells which was unaffected by IgRT.

**Table 4:** T cell subpopulations in patients with IgG subclass deficiency.

	Controls		IgG subclass deficiency			
			All off IgRT		All on IgRT	
	(n=34)		(n=30)		(n=30)	
	Median	IQR	Median	IQR	Median	IQR
T helper subpopulations (% of CD4^+^ T cells)						
Naïve CD45RA^+^CCR7^+^	37	31-49	36	25-50	34	23-50
CM CD45RA^-^CCR7^+^	33	27-44	39	29-52	40	28-50
EM CD45RA^-^CCR7^-^	18	14-25	17	11-22	16	9-28
TEMRA CD45RA^+^CCR7^-^	0.5	0.4-2.2	0.8	0.4-3.0	0.7	0.3-3
Senescent CD28^-^	1.9	0.4-7.9	1.4	0.4-6.1	1.8	0.3-8.7
Activated HLA-DR^+^	3.7	3.1-4.9	2.7**	1.7-3.8	3	2.2-4.5
T cytotoxic subpopulations (% of CD8^+^ T cells)						
Naïve CD45RA^+^CCR7^+^	18	15-30	19	11-35	15	11-36
CM CD45RA^-^CCR7^+^	5.9	3.8-11	7.1	4.4-12	4.5	2.5-9.7
EM CD45RA^-^CCR7^-^	34	28-48	31	18-49	42	22-55
TEMRA CD45RA^+^CCR7^-^	29	15-45	22	13-40	21	13-34
Senescent CD28^-^	33	21-47	31	19-48	34	18-49
Activated HLA-DR^+^	7.8	5.5-10	4.7*	2.8-7.7	4.5	3.5-7.5
Treg populations (% of CD4^+^ T cells)						
Resting CD45RA^+^CD25^low^	0.9	0.6-1.2	0.7*	0.3-1.0	0.7	0.4-0.9
Activated CD45RA^-^CD25^high^	2.9	2.1-3.6	1.4***	0.9-2.0	1.7	1.3-2.5

IQR, inter quartile range; CM, central memory; EM, effector memory; TEMRA, terminal effector memory RA^+^; ^*^p<0.05; ^**^p<0.01; ^***^p<0.001, compared to controls. Wilcoxon paired test was used for comparisons between IgGsd patients off and on therapy, while Mann-Whitney U-test was used for non-paired analysis.

### Reduced regulatory T cells in patients with IgG subclass deficiency

**Figure 4:**
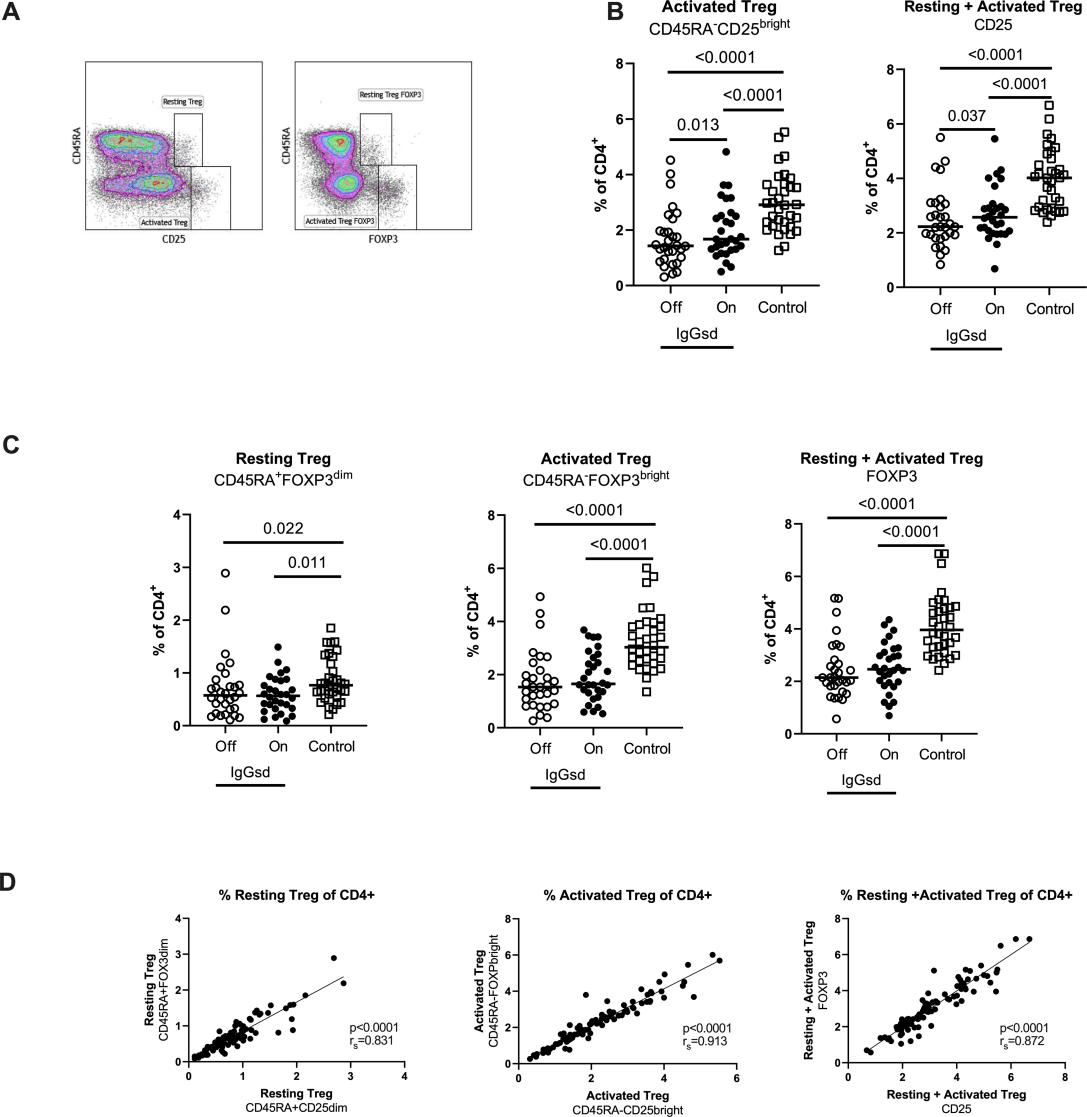
Decreased activated regulatory T cells (Tregs) in IgG-subclass deficiency patients is partly restored during Ig replacement therapy. Frequencies of Tregs were assessed by flow cytometry using two different strategies: cell surface expression of CD25 or intra cellular expression of FoxP3, and the activation status of Tregs was defined by the expression of CD45RA **(A)**. Lower levels of activated Tregs in IgG subclass deficiency (IgGsd) patients (open circle) were partly restored during (filled circle) immunoglobulin replacement therapy (IgGRT) when compared to healthy controls **(B)**. Frequencies of Resting, activated, and resting + activated Tregs defined by FoxP3 and CD45RA expression showed a pattern similar to Tregs defined by CD25 and CD45RA **(C)**. There was a strong correlation between frequencies of Treg populations defined by CD25 and FoxP3, respectively **(D)**. Wilcoxon paired test was performed between IgGsd patients Off and On immunoglobulin replacement therapy, while Mann-Whitney U-test was used for non-paired analysis, and Spearman (r_s_) for correlation analysis.

Further, we found reduced levels of CD4^+^ Tregs in patients with IgGsd compared to controls (**Table 4**) and this finding was mainly related to the activated CD45RA^-^CD25^bright^ Treg subset (**Figures 4A, B**). There was a high compliance between this gating strategy for Tregs and an alternate gating strategy that included the transcription factor Foxp3 with Spearman correlation (r_s_) 0.91 for activated Treg cells (**Figures 4C, D**). When patients were on IgRT, the Treg compartment expanded within the Th cell population, mainly due to increased proportions of activated Treg cells, but the levels were still lower compared with healthy controls (**Figure 4A**). We found no association between activated Tregs and the presence of autoimmune conditions in patients with IgGsd. In summary, IgGsd was characterized by decreased activated and total Tregs, which were partly restored during IgRT.

### Increased plasma levels of costimulatory and regulatory immune checkpoint molecules in patients with IgG subclass deficiency

**Figure 5:**
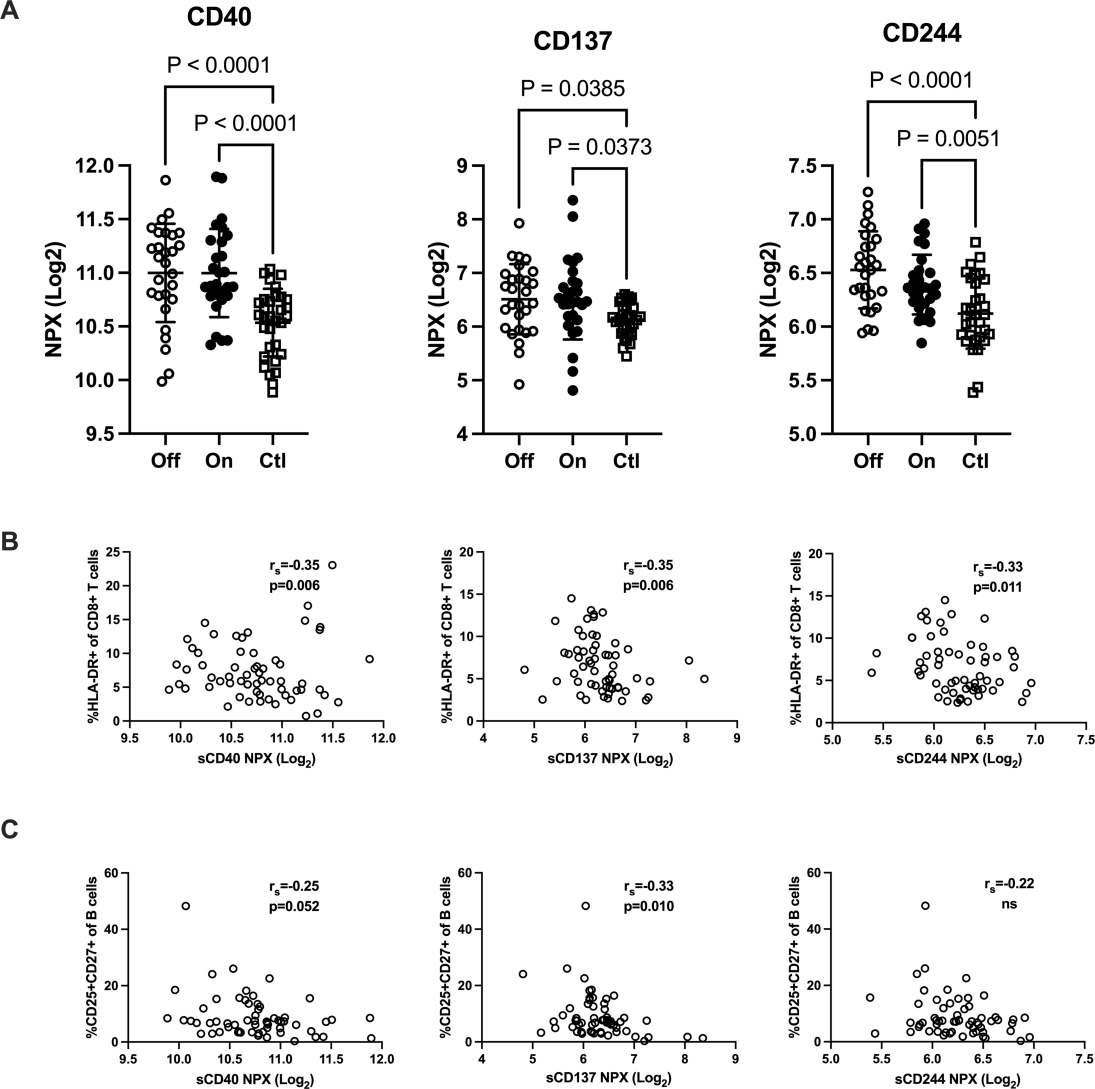
IgG-subclass deficiency is associated with increased plasma levels of checkpoint molecules. Plasma levels of T cell response regulating factors CD40, CD137 (TNFRSF9) and CD244 were analyzed with proximity extension assay in patients with IgG-subclass deficiency (IgGsd) when off and on Ig-replacement therapy (IgRT) compared to healthy controls **(A)**. Correlation of T cell response regulating factors with activated CD8^+^ T cells **(B)** and activated memory B cells **(C)**. Data is presented as normalized protein expression (NPX) values in Log2 scale. Error-bars indicate 1SD, p represent ANOVA with Tukey posttest and Spearman correlation (r_s_), respectively.

Shedded transmembrane proteins can be detected in plasma and provide additional aspects of the immune function in patients with IgGsd. Circulating levels of transmembrane receptors were analyzed in samples collected from 28 patients when on and off IgRT and compared to cross-sectional healthy control samples. In our dataset, there were 18 proteins with predominant location in the plasma membrane. Sixteen of them were detected in more than 90% of samples and selected for further analysis (**Supplementary Table S2**). Whether the patients had ongoing IgRT or had no therapy, did not affect the plasma levels of any factor. Plasma levels of CD40, CD137 and CD244 were elevated in patients with IgGsd, both when on and when off IgRT, compared to controls (**Figure 5A**). These proteins regulate immune responses, and soluble isoforms of CD40 and CD137 are secreted by various cell types including immune cells (28, 29). Circulating levels of soluble CD40, CD137 and CD244 all showed a moderate correlation with activated HLA-DR^+^ CD8^+^ T cells (**Figure 5B**). Moreover, soluble CD137 correlated moderately and soluble CD40 correlated weakly with activated CD25^+^ memory B cells (**Figure 5C**). In addition, levels of CD6, CD215 (interleukin, IL-15 receptor A), CD218 (IL-18 receptor 1) and CD318, a ligand for CD6 also known as CDCP1 (for CUB domain-containing protein 1) were elevated in IgGsd patients in samples collected when on IgRT (CD218) or when off IgRT (CD6, CD215 and CD318) (**Table 5**). All these proteins are membrane bound, but CD6, CD215 and CD318 are also produced as soluble molecules (30–32). Taken together, the elevated levels of proteins involved in immune responses such as immune checkpoints associated with T and B cell phenotypes in IgGsd.

**Table 5:** Elevated immune checkpoints in plasma of patients with IgGsd.

	HC (n=32)			IgGsd (n=28)		
				IgRT		No IgRT
Factor	Mean (NPX)	SD	Mean (NPX)	SD	Mean (NPX)	SD
CD6	4.53	0.46	4.88	0.68	4.91*	0.66
CD215	0.69	0.32	0.84	0.35	0.92*	0.40
CD218	7.62	0.40	7.98*	0.57	7.90	0.47
CD318	2.02	0.49	2.48	0.80	2.55*	0.95

*p<0.05 compared to HC, ANOVA. NPX, normalized protein expression in Log_2_ scale; Uniprot, Uniprot-id; HC. healthy controls; IgGsd, immunoglobulin subclass deficiency; IgRT, immunoglobulin replacement therapy; SD, standard deviation.

## Discussion

In this prospective study we have compared the adaptive arm of the immune system in patients with IgGsd, on and off subcutaneous IgRT, with a group of cross-sectional healthy controls. The study protocol required at least 6 months of IgRT discontinuation before considering patients as being off IgRT to ensure that residual IgRT not was captured in laboratory testing. IgG3 deficiency was the most common finding in our cohort, either alone or in combination with other IgG-subclass deficiencies. Only 23% and 43% of patients were diagnosed with IgG1 and IgG2 subclass deficiency, respectively, which may explain why IgG1- and IgG2 median values were within the reference intervals based on the 2.5 – 95 percentiles of a healthy population (33). Serum levels of IgG4 were not tested since IgG4 constitute less than 5% of total serum IgG and is of minor importance in infection defense (34). In addition, the lower reference limit (2.5 percentile) of IgG4 is 0.05g/L which makes the clinical relevance of an isolated IgG4sd uncertain (33).

Most patients with IgGsd had one or several comorbidities such as autoimmunity, atopy, or reduced lung function. We found no connection with any of the comorbidities and more complex IgG subclass deficiencies or subnormal levels of IgG and IgA. Overall, the IgGsd patients in this cohort shared clinical characteristics with other cohorts of patients with different forms of IgGsd (6, 7, 10). Absolute numbers of B cells, NK cells, Th cells and cytotoxic T cells did not differ between IgGsd patients and healthy controls and were not affected by IgRT, which is in line with previous reports of minor PAD (10, 17, 35). Deeper analysis of the B cell and T cell subsets in our study showed that activated (CD25^+^) memory B cells were lower in IgGsd and normalized during IgRT, which might reflect the important role of antibodies in B-cell activation. Low frequencies of switched memory B cells are common in CVID and correlate with lymphoproliferation (15, 36). Only two of the patients had subnormal switched memory B cells, suggesting that the underlying pathophysiology of IgGsd may differ from other more severe PAD in which low switched memory B cells is a common finding such as CVID and CVID-like disorders.

The activation status of Th cells and cytotoxic T cells was lower in IgGsd compared to controls. While on IgRT, the effector memory subset of CD8^+^ T cells increased, which might reflect an improvement of T cell function. This is in line with the reduced infection burden in patients with IgGsd treated with IgRT (6).

Compared to controls, decreased frequencies of Treg subsets were a consistent finding in IgGsd both while on and off IgRT. This is a novel finding in IgGsd, and its importance remains to be determined. Tregs suppress immune responses by cellular interactions with antigen presenting cells, and modulation of the local environment by the secretion of immune inhibitory cytokines and deprivation of immune stimulatory cytokines (37). Most autoimmune diseases display defects in either the number or function of Tregs (18). Lower Tregs could contribute to the development of autoimmunity and atopic disease in patients with IgGsd. Also, bronchiectasis can be considered a chronic inflammatory disease (38), which may worsen by impaired Treg function. When phenotyping Treg cells we used an appropriate strategy that avoids inclusion of false positive Foxp3 cells (37), and we found a good correlation between alternative gating strategies. Tregs have been extensively studied in CVID, where decreased proportions of Treg cells are considered to be associated with an increased risk of autoimmune manifestations (39), even if some conflicting data exist (40). In our cohort, we did not find a clear association between Treg frequency and autoimmunity, but this needs to be further explored in future studies of IgGsd. In CVID it has been demonstrated that IgRT transiently increases the number of Tregs immediately after infusion (41), but a long-term follow-up trial did not show any effect of IgRT on the Foxp3+ regulatory T cell compartment in CVID (24). Our observation, that the Treg compartment was only partially restored during IgRT may relate to the ability of IgG to act as an effector molecule as well as a regulatory molecule. There are accumulating data that high-dose intravenous immunoglobulin therapy increases the number of circulating Tregs and their immunosuppressive capacity in many inflammatory and autoimmune conditions (42). Based on these findings, a more significant effect on the Treg compartment in IgGsd during IgRT would have been expected. However, the use of lower doses and that subcutaneous administration instead of intravenous, as well as chronic longstanding inflammation rather than an acute inflammatory condition in IgGsd and other PAD, make any comparison difficult.

Increased levels of circulating costimulatory and regulatory immune checkpoints molecules, may contribute to the aberrant B- and T cell subpopulations found in patients with IgGsd. Both CD40 and CD137 belong to the TNF receptor superfamily and are key regulators of adaptive immune responses. CD40 is expressed not only by immune cells but also by epithelial and endothelial cells (29). Soluble (s) CD40 results mainly from shedding (43) and can act as competitor of the interaction of membrane CD40 with its ligand CD154 and negatively affect B cell activation and T cell dependent antibody responses (29). Soluble CD137 is secreted by activated T cells and encompasses the whole extracellular domain (44). Hence, increased sCD137 may counteract CD137 and CD137 ligand interactions, hampering T cell activation (28) and T cell dependent antibody responses (45). Negative correlations between sCD40 as well as between sCD137 and the proportions of activated memory B cell and activated cytotoxic T cells in this study may reflect inadequate immune suppression in IgGsd, which may confer impaired immune responses and increased susceptibility to infections. CD244 is a transmembrane receptor present mainly in NK cells and T cells, which provides stimulatory and inhibitory signals, regulate cytotoxic cellular immune responses. To date, there are no evidence for soluble forms of CD244. However, increased plasma levels of CD244 have been reported in hepatitis C infection (46). The elevated levels of sCD244 in the present study could reflect increased shedding and/or turnover of T cells and NK cells.

CD6, CD215, CD218 and CD318 were other dysregulated transmembrane receptors in patients with IgGsd. CD6 is a pattern recognition receptor expressed by most T cells, which is released into circulation by proteolytic cleavage (46). CD318 is widely expressed by epithelial cells and is a ligand for CD6 (32). CD6 has an inhibitory effect on T cell responses (47). Increased levels of sCD215 (IL-15RA) have been reported in synovial fluid of patients with rheumatoid arthritis (48). Membrane CD218 (IL-18R1) is involved in activating T helper 1 responses (49) but knowledge about how sCD218 may affect immune responses is missing. CD218 was the single factor only elevated in samples collected when on IgRT. In summary, increased levels of sCD40 and sCD137 may contribute to the decreased activation of B cells and T cells found in patients with IgGsd and it can be hypothesized that sCD40 and sCD137 impair immune responses and the secretion of IgG in IgGsd.

In a previous study, where we investigated a large number of secreted cytokines in plasma of patients with IgGsd, levels were unaffected by IgRT (25). Together these findings indicate no anti-inflammatory effects of IgRT in IgGsd. Instead, we found that CD4^+^ T cell activation and B cell activation were increased, and partly restored, as well as increased effector memory CD8^+^ T cells in patients when on IgRT. These findings imply that IgRT may augment the adaptive arm of the immune response in IgGsd. Increased soluble checkpoints both when on and off IgRT, may be driven by underlying inflammatory conditions or tissue damage not affected by IgRT. However, increased soluble immune checkpoints and aberrations in B- and T cell subpopulations clearly are indicatives of an underlying immune dysfunction in patients with IgGsd.

The prospective design and the paired samples for comparing immunological phenotypes when the patients were on and off IgRT are strengths of this study. The low number of patients enrolled in the study is a limitation, and the findings need to be verified in other larger cohorts. The heterogeneity of patients with deficiencies of different IgG subclasses and their different combinations of comorbidities makes it difficult to draw any general conclusions from this cohort. Using severe lung disease as an exclusion criterion may also have impact on the results, since patients with manifest lung disease are the ones that benefits most from IgRT.

In summary, numbers of B cells, T cells and NK cells were similar in patients with IgGsd and healthy controls. Deeper analysis revealed decreased activation of CD4^+^ and CD8^+^ T cells, and memory B-cells in patients with IgGsd. The findings were consistent with negative correlation between plasma levels of immune checkpoint molecules and the levels of CD8^+^ T cell and memory B cell activation. The activation of T cells and immune checkpoint levels were not affected by IgRT, but the activation of B memory B cells was partly restored. Activated Tregs were reduced in patients with IgGsd, and partly restored during IgRT. Hence, IgRT may contribute to improved immunity in patients with IgGsd. The significance of the association between low activated Tregs and IgGsd remains unclear and should be further investigated in future studies.

## Data Availability

The original contributions presented in the study are included in the article/**Supplementary Material.** Further inquiries can be directed to the corresponding author.
