# Supplementary material for: Immunological characterization of IgG subclass deficiency reveals decreased Tregs and increased circulating costimulatory and regulatory immune checkpoints
Source: Front Immunol. 2024 Aug 14;15:1442749. doi: 10.3389/fimmu.2024.1442749 (PMC11349633; doi:10.3389/fimmu.2024.1442749)
Supplement: Supplementary file 1 [file DataSheet1.pdf]

Immunological characterization of IgG subclass deficiency reveals decreased Tregs and increased circulating costimulatory and regulatory immune checkpoints

Per Wågström[1,2*], Maria Hjorth[3], Daniel Appelgren[4], Janne Björkander[5], Charlotte Dahle[3], Mats Nilsson[6], Åsa Nilsdotter-Augustinsson[2], Jan Ernerudh[3] and Sofia Nyström[3]

Supplementary Information Tables

Table S1. Monoclonal antibodies used for flow cytometry

Monoclonal antibody	Conjugation	Clone	Company
CD3/8/45/4	FITC/PE/PerCP/APC		BD
CD3/16+56/45/19	FITC/PE/PerCP/APC		BD
CD45RA	FITC	L48	BD
CD24	FITC	ML5	BD
CD3	PE	UCHT1	BD
CD27	PE	L128	BD
Foxp3	PE	PCH101	eBioscience
CD38	PerCP Cy5.5	HIT2	BD
HLA-DR	PerCP	G46-6	BD
CD4	PE-Cy7	SK3	BD
CD25	PE-Cy7	M-A251	BD
CD28	APC	CD28.2	BD
CD21	APC	B-ly4	BD
CD4	APC	SK3	BD
CD8	APC-H7	SK1	BD
IgD	APC-H7	IA6-2	BD
CCR7	Horizon V450	150503	BD
IgM	Horizon V450	G20-127	BD
CD3	Horizon V450	UCHT1	BD
CD27	Horizon V500	M-T271	BD
CD19	Horizon V500	HIB19	BD

Table S2 Detection levels and linearity of Olink Target Inflammation panel*

Transmembrane receptor	*Uniprot*	*Range in study population (NPXLog2)* min	max	*LLOD (NPXLog2)*	*Call rate (%)*
CD8A	P01732	8.034	11.60	-0.193	100
CD318	Q9H5V8	0.953	4.904	-0.820	100
CD244	Q9BZW8	5.387	7.255	1.307	100
OPG	O00300	9.093	11.259	-0.004	100
IL-20RA	Q9UHF4	-0.134	1.608	0.484	42
IL-2RB	P14784	-0.122	1.706	0.613	51
CD6	P30203	3.267	6.825	-0.331	100
SLAMF1	Q13291	0.149	1.668	0.406	90
IL-10RA	Q13651	-0.401	2.650	0.021	76
LIF-R	P42702	2.520	3.607	0.162	90
CD215	Q13261	-0.019	1.669	-0.243	100
IL-10RB	Q08334	3.629	5.718	0.236	100
IL-22RA1	Q8N6P7	0.124	3.208	1.169	100
CD218	Q13478	6.499	9.046	0.775	100
PD-L1	Q9NZQ7	5.216	7.908	1.541	100
CD5	P06127	3.047	5.383	-0.932	100
CD40	P25942	9.887	11.895	0.474	100
CD137	Q07011	4.809	8.354	1.009	100

* Validation data reported by Olink. based on samples spiked with recombinant proteins. Uniprot, uniport-id. LLOD, lower limit of detection; NPX, normalized expression.
